# A Novel Wearable Device for Continuous Blood Pressure Monitoring Utilizing Strain Gauge Technology

**DOI:** 10.3390/bios15070413

**Published:** 2025-06-27

**Authors:** Justin P. McMurray, Aubrey DeVries, Kendall Frazee, Bailey Sizemore, Kimberly L. Branan, Richard Jennings, Gerard L. Coté

**Affiliations:** 1 Department of Biomedical Engineering, Texas A&M University, College Station, TX 77843, USA; aubreydevries@tamu.edu (A.D.); kendallfrazee@tamu.edu (K.F.); baileysizemore@tamu.edu (B.S.); klb4333@tamu.edu (K.L.B.); richard.jenningii@tamu.edu (R.J.); gcote@tamu.edu (G.L.C.); 2 Texas A&M Engineering Experiment Station, Center for Remote Health Technologies and Systems, College Station, TX 77843, USA

**Keywords:** continuous blood pressure monitoring, wearable biosensor, strain gauge technology, cuffless blood pressure, hypertension management, noninvasive sensor technology

## Abstract

Cardiovascular disease (CVD) is the leading cause of global mortality, with hypertension affecting over one billion people. Current noninvasive blood pressure (BP) systems, like cuffs, suffer from discomfort and placement errors and lack continuous monitoring. Wearable solutions promise improvements, but technologies like photoplethysmography (PPG) and bioimpedance (BIOZ) face usability and clinical accuracy limitations. PPG is sensitive to skin tone and body mass index (BMI) variability, while BIOZ struggles with electrode contact and reusability. We present a novel, strain gauge-based wearable BP device that directly quantifies pressure via a dual transducer system, compensating for tissue deformation and external forces to enable continuous, accurate BP measurement. The reusable, energy-efficient, and compact design suits long-term daily use. A novel leg press protocol across 10 subjects (systolic: 71.04–241.42 mmHg, diastolic: 53.46–123.84 mmHg) validated its performance under dynamic conditions, achieving mean absolute errors of 2.45 ± 3.99 mmHg (systolic) and 1.59 ± 2.08 mmHg (diastolic). The device showed enhanced robustness compared to the Finapres, with less motion-induced noise. This technology significantly advances current methods by delivering continuous, real-time BP monitoring without reliance on electrodes, independent of skin tone, while maintaining a high accuracy and user comfort.

## 1. Introduction

CVD is the leading cause of death globally [1]. Hypertension is a significant risk factor for CVD as it can cause damage to arteries, heart strain, the formation of aneurysms, kidney damage, heart failure, heart attack, and stroke [2]. In the United States, there are also notable disparities in the incidence and management of CVD and hypertension, with a higher incidence and effect in underserved, often underrepresented, populations with varying skin tones [3]. Managing high BP through lifestyle changes, medication, and regular monitoring is crucial in reducing the risk of developing or worsening the effects of CVD and hypertension [4].

Invasive arterial lines are the gold standard for continuous BP measurement, while cuff-based BP systems are the standard for noninvasive and non-continuous BP measurements [5]. While auscultatory measurements are also standard, it is often not practical to have trained medical staff, who are required to perform the measurement, anywhere a patient needs their BP measured [6]. Oscillometric methods encompass automatic and manual BP and cuff-based monitors, which are more commonly used but are cumbersome and have limitations and inaccuracies [7,8]. For example, blood pressure accuracy is reduced when the device is misaligned with the artery; if the subject talks, shifts positions, or contracts their muscles during the measurement; or if they have not rested for at least 5 to 25 min prior to taking the measurement [9,10,11]. This method is also subject to white coat hypertension, where BP levels are elevated due to the subject’s anxiety related to taking the measurement [12]. There is also a need to wait 1 to 2 min between cuff-based measurements to allow blood vessels to return to their normal state, reducing the potential for falsely elevated readings due to residual pressure or compression from the previous measurement [13]. The time delay prevents cuff-based systems from having the ability to monitor BP continuously [14]. These limitations require noninvasive, continuous BP monitoring solutions that overcome motion artifacts, recalibration requirements, and inaccuracies across diverse populations.

Noninvasive biomedical devices that measure continuous BP for managing hypertension and reducing CVD-related ailments are emerging to overcome the problems with cuff-based systems [15]. Standard commercial-grade wearable sensors in smartwatches, smart rings, and fitness trackers often include PPG technology [16]. As a result, this modality has been used in an attempt to quantify BP through indirect correlation, since it is already embedded within existing form factors [17]. Accurate BP measurements often require calibration against a standard method, and variability in the calibration can impact the reliability of continuous monitoring [18]. The primary problem with using PPG measurements to monitor BP is that they can often be less accurate than traditional cuff-based BP measurements due to the inherent properties of the sensor [19]. PPG sensors are susceptible to motion artifacts, exhibit a lower accuracy and sensitivity than other noninvasive methods, and are influenced by external factors such as skin tone and body mass index (BMI), making them unreliable for a broad range of users (Table 1). PPG sensors are sensitive to noise artifacts from movement, skin contact pressure, variations in skin tone, variability among individuals’ vasculature, body mass index, and other physiological factors that make it a problematic modality to scale across diverse populations [20,21]. Location on the body and even local changes in position can change the morphology of the captured signal, amplitude, and the signal’s DC offset, making it difficult to obtain consistent, reliable BP measurements under dynamic conditions [15,22,23]. Sophisticated algorithms and signal-processing techniques have helped overcome some of these issues [24]. However, applying these techniques to user-friendly wearables with an acceptable accuracy remains challenging due to the complexities involved. Other noninvasive wearable sensors for BP monitoring include BIOZ, epidermal strain sensors, radar and microwave sensors, and acoustic sensors, where some utilize pulse transient time (PTT) to calculate BP, the Doppler Effect, and other methods such as monitoring morphology and sensor response [25,26,27,28]. Like the PPG sensor, BIOZ requires regular recalibration and is influenced by BMI (Table 1), making long-term continuous monitoring difficult [29]. BIOZ is sensitive to noise artifacts such as movement, electrode placement, skin temperature, tissue composition, electrode quality, durability, and manufacturability [30,31,32]. Additionally, BIOZ has challenges obtaining real-time and accurate BP readings due to the need for complex adaptive algorithms [25]. Acoustic devices also struggle with accuracy, require frequent recalibration, and are highly sensitive to motion artifacts, further limiting their practicality for real-world use (Table 1). Epidermal strain sensors require precise calibration and placement, while individual differences can make calibration difficult [33]. Long-term adhesion can be difficult and cause skin irritation, motion sensitivity, and slow delamination of the flexible substrate, which will cause a drift in the BP calibration, all of which are affected by environmental factors like temperature, humidity, and water exposure [34,35,36]. Radar and microwave sensors overcome comfort and skin irritation. However, they are sensitive to electromagnetic interference, have high power requirements that can limit the potential for continuous monitoring, require complex algorithms, and have high costs to support the technology and technical complexity of the equipment [37,38,39].

Unlike these existing methods, strain gauge-based BP monitoring technology offers a high accuracy and sensitivity while being resistant to common issues such as skin tone variations and BMI influence (Table 1). The technology is well-suited for continuous monitoring and does not require frequent recalibration, making it an optimal choice for real-world applications. The limitations of commercial- and research-grade devices show that the need persists for a device to accurately and continuously measure BP in a form factor that would be adaptable by users and remain affordable and, therefore, accessible, as shown in Table 1 [15].

**Figure 1:**
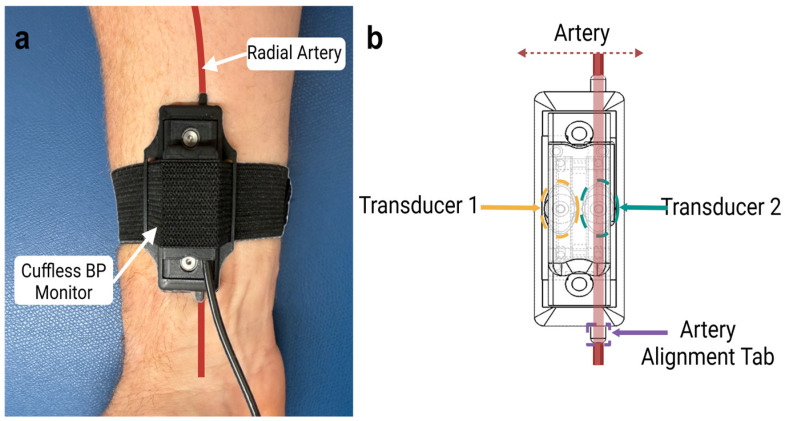
Cuffless blood pressure monitor. (**a**) Cuffless blood pressure monitor placed on the wrist with either transducer on or near the artery. (**b**) Diagram of cuffless blood pressure monitor showing placement of the primary transducer 2 that captures the arterial pulse pressure and reference transducer 1, along with their relative alignment.

To overcome the many issues associated with the above BP monitoring approaches, in this study, a wearable device that integrates a novel strain gauge technology into a reusable wearable sensor consisting of two pressure transducers for direct BP quantification was designed, built, and tested (Figure 1). This long-term reusable, comfortable device is suitable for extended wear (e.g., 24 h) due to its strap-based design eliminating the need for adhesive materials. We chose this method as it is agnostic to skin tone, makes it easy to integrate strain gauges into existing form factors, has a lower cost of materials, has less need for complex algorithms due to direct pressure measurements, and has lower power consumption requirements than PPG and BIOZ, allowing an extended device life before needing to be recharged [50,51].

The wearable is initially calibrated to actively monitor skin contact pressure, while the pressure response is used to estimate BP [50]. During data collection, the wearable is first placed on the wrist above the radial artery, and subjects are measured in static positions where we vary the skin contact pressure [52]. This allows us to characterize the system response and derive corrections for contact pressure variations.

**Figure 2:**
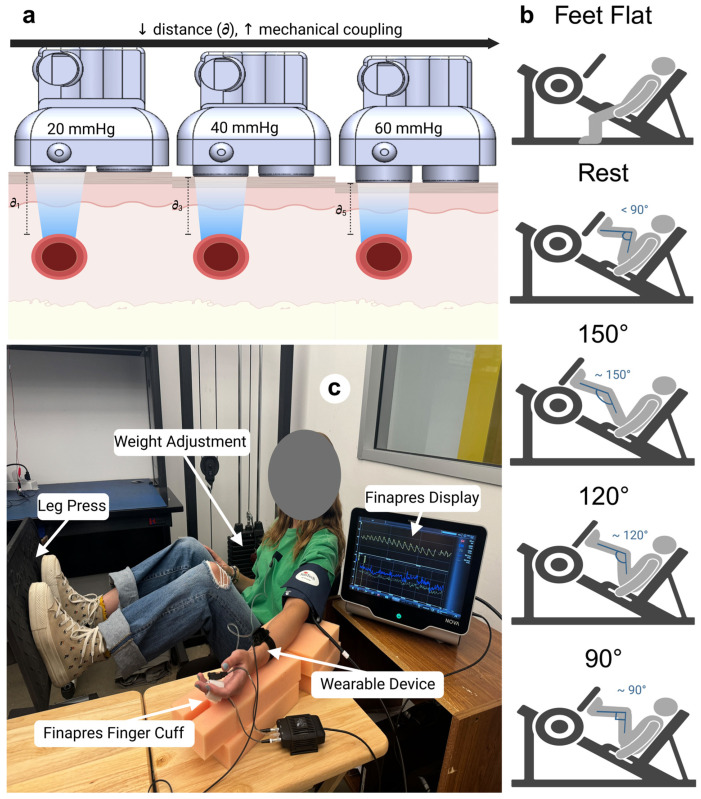
Test setup and device configuration for testing cuffless blood pressure monitoring. (**a**) The schematic of the device shows how extending the transducer increases contact pressure and mechanical coupling and reduces the distance between the device and the artery. (**b**) Illustration of leg angles for dynamic testing on a leg press machine, with positions at 90°, 120°, 150° and a resting and feet-flat position. These angles simulate varying exertion levels to assess the device’s accuracy for changing BP. (**c**) Setup of the wearable device on the participant’s forearm, with artery alignment marks for optimal placement. The finger cuff is connected to the Finapres Nova, providing continuous reference BP measurements during testing.

To evaluate the dynamic range of BP measurements collected by our device, participants followed a unique protocol to utilize a leg press at four marked positions to induce changes in their BP (systolic: 71.04–241.42 mmHg, diastolic: 53.46–123.84 mmHg) [53] (Figure 2) Our device accurately captured BP under dynamic conditions during the trials, with a mean absolute error (MAE) of 2.45 ± 3.99 mmHg for systolic and 1.59 ± 2.08 mmHg for diastolic BP.

While these results demonstrate the potential of the device for noninvasive, continuous BP monitoring, this study does not aim to establish compliance with specific regulatory standards. Instead, our findings provide a foundation for future studies that may involve larger sample sizes and formal clinical validation under standardized protocols.

## 2. Materials and Methods

### 2.1. Human Subject Recruitment

The study cohort consisted of ten adults (seven males and three females) aged 20 to 40. Physiological data was collected under a rigorously approved protocol, with the primary objective of evaluating exercise-induced BP variations.

The cohort included participants with diverse skin tones (six light, two medium, two dark), though this distinction was deemed irrelevant due to the device’s reliance on mechanical movement rather than optical or photoplethysmographic methods. While body mass index (BMI) was not explicitly tracked, all participants were confirmed to be in a healthy physiological state [54].

### 2.2. Design and Fabrication

**Figure 3:**
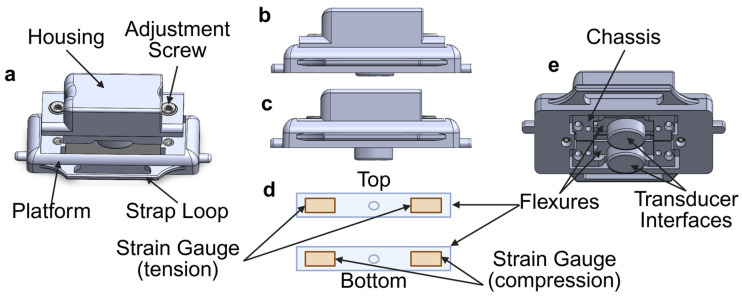
Multiple views of the cuffless blood pressure monitoring device’s design. (**a**) Top view displaying the device’s housing, adjustment screw, platform, and strap loop. The design allows secure attachment and precise alignment with the radial artery. (**b**) Side view with the transducer interface probe fully contracted. (**c**) Side view with the transducer interface probe fully extended, showing the range of adjustment for optimizing arterial contact. (**d**) Internal layout of the top and bottom flexures, with strain gauges in tension and compression configurations to detect arterial deformation. (**e**) Bottom view displaying the device’s transducer interfaces, flexures, and chassis, engineered for stability and reliable arterial pressure monitoring.

The cuffless BP monitoring device was designed using SOLIDWORKS (2023) to enable the precise modeling of its components. Individual parts were fabricated using 3D printing with Polylactic Acid (eSun PLA Pro, Shenzhen eSUN Industrial Co., Shenzhen, China) filament and the 3D printer (Bambu Lab X1-Carbon, Shenzhen, China). This setup was selected for its high accuracy, efficiency, and ease of use, which were critical for rapid prototyping and iterative design during the development process. As shown in Figure 3, the device features a robust housing and a sturdy chassis, with dual flexures integrating fixed transducer interfaces. An adjustable moving platform equipped with shoulder screws, bearings, and threaded inserts allows for precise control over transducer contact pressures. The flexures were empirically optimized to achieve the required dynamic range and sensitivity, ensuring accurate pressure measurements under the intended operational parameters. The final assembled device measures approximately 55 mm in length, 25 mm in width, and 11 mm in height.

Each flexure is configured in a full Wheatstone bridge, incorporating four 350 Ω strain gauges (Micro-Measurements N2A-06-S5150R-10C/E4, Micro-Measurements, Raleigh, NC, USA) to enhance the sensitivity and linearity of the pressure response. This setup ensures the high-fidelity measurements crucial for the device’s accuracy and reliability. The strain gauge bridges are powered by a 3 V supply, with output signals routed to a dual-channel 24-bit analog-to-digital converter (ADC) (Nuvoton NAU7802, Nuvoton Technology Corporation, Hsinchu, Taiwan). The ADC has a programmable gain amplifier (PGA) to optimize signal integrity and reduce noise during measurement.

Data acquisition is handled through a microcontroller (STM P-NUCLEO-WB55, STMicroelectronics, Geneva, Switzerland), which interfaces with the ADC using Inter-Integrated Circuit (I2C) communication for real-time data processing. The processed data is then transmitted to a local computer via the Universal Asynchronous Receiver/Transmitter (UART) protocol, where it is analyzed and visualized using MATLAB 2024a software(MathWorks, Natick, MA, USA). Real-time signal visualization was crucial for qualitative verification during testing, ensuring accurate placement of the device and optimal contact pressure.

### 2.3. System Calibration 

The cuffless BP monitoring system was initially calibrated by placing the device flush on a flat, calibrated surface. Both transducers were aligned properly to ensure uniform contact and optimal performance. Calibration weights of varying magnitudes—10, 20, 50, 100, 200, and 500 g—were utilized to span the device’s operational range.

Each weight was centrally positioned during calibration in the x and y axes atop the device, ensuring a uniform weight distribution across the two transducers. The contact surface area of each ellipse transducer was used to convert the applied weight from grams to mmHg according to the following relationship:

(1) $$P_{mmHg}=\frac{mg}{\left(\pi ab\right)*133.3}$$

where m is mass, g is gravity, a is the length of the major axis, b is the length of the minor axis, and 133.3 is the conversion from Pascal to mmHg.

Analog-to-digital converter (ADC) values were recorded for each weight placement, establishing a dataset for calibration analysis. A linear calibration curve was generated for each transducer, allowing for the precise mapping of ADC values to corresponding pressure readings. This calibration model was subsequently programmed into the device’s microcontroller, enabling a real-time pressure display, recording, and response visualization in MATLAB, expressed in mmHg.

The intentional misalignment of the two pressure sensors is a critical component of the device’s function, ensuring an accurate BP estimation by accounting for external contact pressure and tissue mechanics. The primary transducer captures the arterial pressure waveform, while the reference transducer, placed off the artery, measures external contact pressure. The pressure difference between these two sensors is directly incorporated into the calibration model, allowing for the dynamic compensation for variations in tissue compression, sensor movement, and external forces. This differential measurement approach minimizes the artifacts introduced by changes in contact pressure and ensures that the extracted arterial pressure signal remains accurate, regardless of slight variations in sensor positioning. By explicitly integrating the measured pressure difference into the BP estimation model, the system eliminates the need for additional correction due to misalignment, enhancing the calibration’s robustness and improving real-time BP monitoring accuracy.

Daily calibration checks were performed before data collection, utilizing the calibration procedure to ensure the high accuracy and reliability of pressure measurements throughout the experimental phase. While these daily checks were implemented to detect any potential sensor drift and eliminate additional sources of error in our research setup, they would not necessarily be required in a finalized consumer device built for average users. The initial calibration model would provide a sufficient accuracy for typical use without ongoing adjustments. However, we recorded these daily values to confirm that the device maintained a consistent accuracy in the contact pressure and pressure response readings during each experimental session.

### 2.4. Transducer Calibration and Characterization

An additional set of small weights—specifically 1, 2, 4, and 5 g—was applied following the initial calibration to further characterize the system’s sensitivity and confirm its resolution. The elliptical contact area of the transducers was measured at approximately 62.53 mm^2^, based on dimensions of 11.89 mm by 6.7 mm. The smallest weight, 1 g, corresponded to a theoretical pressure of approximately 1.17 mmHg over this area. The system detected this as 1.23 mmHg, with a minimal deviation (0.06 mmHg) attributed to calibration factors or inherent system noise, affirming the device’s capability to accurately detect changes around 1 mmHg.

However, the system’s effective resolution is better demonstrated by its ability to measure subtle changes in arterial pressure waveform amplitude. By analyzing the device’s AC amplitude (defined as the difference between the pulse waveform peak and foot), we observed that the system could detect variations as small as 0.0006 mmHg. This sensitivity enabled the system to capture clear, detailed pulse waveforms, revealing subtle changes between 0 and 0.0006 mmHg. Based on the 24-bit ADC and full measurement range, the theoretical resolution was calculated at 0.0000143 mmHg, while practical limitations, including noise, set an effective resolution well below 0.0006 mmHg.

This characterization underscores the system’s ability to reliably detect minute changes in BP across the 0 to 240 mmHg range. These findings confirm that the device’s high sensitivity and precise measurement capabilities are well-suited for continuous and accurate BP monitoring applications where nuanced pressure detection is critical.

### 2.5. Physics

The dual-pressure transducer system for cuffless BP measurement relies on capturing arterial pressure at the radial artery while compensating for tissue deformation and external forces. One transducer is placed over the radial artery, and the other is positioned parallel but displaced to measure the contact pressure from tissue. The pressure waveform in the artery P_α_(t) can be expressed as a function of time and includes harmonics from the heart’s contraction.

(2) $$P_{\alpha }\left(t\right)=P_{mean}+\sum_{n=1}^{N}A_{n}sin(2\pi f_{n}t+\emptyset_{n})$$

where P_mean_ is the mean arterial pressure, and A_n_, f_n_, and Ø_n_ are the amplitude, frequency, and phase of the nth harmonic component, respectively. The pressure wave is attenuated by the overlying tissue, which can be modeled by

(3) $$P_{measured}(t)=P_{\alpha }\left(t\right)*e^{-\alpha }$$

where P_measured_ is the pressure recorded at the transducer, and α is the attenuation constant based on tissue properties. The attenuation constant α is determined by the mechanical properties of the tissue, including stiffness and density, which vary between subjects.

For transducer 1 (placed over the artery), the measured pressure signal is

(4) $$P_{measured,1}=P_{\alpha }\left(t\right)*e^{{-\alpha }_{1}}+P_{contact,1}(t)$$

where P_α_(t) is the arterial pressure as a function of time, α is the attenuation constant that depends on tissue properties such as elasticity and density, and P_contact,1_(t) is the contact pressure from the tissue pressing against the transducer.

The reference transducer, placed off or near the artery, measures the contact pressure from tissue deformation, and where the measured signal is

(5) $$P_{measured,2}=P_{contact,2}$$

The arterial pressure signal is then isolated by subtracting the contact pressure:

(6) $$P_{\alpha }\left(t\right)=\left(P_{measured,1}-P_{measured,2}\right)*e^{\alpha_{1}}$$

Subjects’ variability due to differences in tissue stiffness, fat percentage, and arterial compliance is accounted for through the attenuation constant α, which is influenced by tissue’s mechanical impedance:

(7) $$Z=\frac{E}{\rho cd}$$

where E is the tissue elastic modulus, ρ is tissue density, c is the speed of sound through tissue, and d is the effective tissue thickness between the artery and the sensor. As tissue stiffness E increases, mechanical impedance Z also increases, creating greater resistance to the pressure wave as it propagates through the tissue, which leads to increased attenuation.

Fat percentage contributes to a larger d, amplifying the exponential decay of the pressure wave and reducing the signal amplitude, even though Z may decrease. Arterial compliance affects pulse waves’ frequency; stiffer arteries generate sharper, high-frequency waves that attenuate more quickly than the smoother, low-frequency waves from more compliant arteries.

### 2.6. Data Collection

#### 2.6.1. Pre-Testing

Prior to data collection, several preparatory steps were taken to ensure accurate and reliable results for each test subject. As mentioned, the device was first calibrated to verify proper functionality. It was then connected to a computer using Arduino and MATLAB interfaces. After establishing the connection, as shown in Figure 2, the device was positioned on the subject’s left radial artery, which was located using Doppler Ultrasound and marked with a surgical marker. The device was placed directly over the marked line and secured with an armband configuration. Proper positioning was confirmed by checking the signal output via the Arduino interface to ensure unobstructed radial artery detection. Next, the Finapres Nova device and its accessories were set up. The Finapres Nova cuff was positioned on the middle segment of the subject’s middle finger, and the device was verified to provide reliable BP signals. Once all the equipment was configured correctly, data collection proceeded systematically according to the testing protocols.

This study involved two types of data collection: static (system characterization) and dynamic testing. Throughout the process, careful attention was given to maintaining consistency and accuracy to ensure the integrity of the results.

#### 2.6.2. Data Collection/Static Testing

In the static testing phase, the subject was seated comfortably in a stationary position, minimizing movement to reduce external variability. The device and the Finapres Nova were positioned on the subject’s left arm and hand, aligned with the radial artery. The contact pressure between the device and the skin was monitored and adjusted using the Arduino and MATLAB interfaces.

We chose a contact pressure range of 20, 30, 40, 50, and 60 mmHg to provide a broad range but also to incorporate realistic levels that mimic the forces exerted by wearable devices such as fitness trackers and smartwatches [55]. Studies indicate that typical wearable devices apply contact pressures in the range of 15–40 mmHg on the skin to maintain stable transducer contact without causing discomfort, ensuring both adequate transducer coupling and user comfort [56]. Additionally, these pressures align with the comfort range typically applied by medical-grade compression socks, which aim to provide support without causing discomfort. Pressures higher than 40 mmHg can become uncomfortable, similar to overly tight compression wear, and are thus less practical for continuous use.

This variation in contact pressure enabled us to systematically assess how tissue compression and mechanical dampening affect the propagation of arterial pulse waves, which are critical for accurate BP measurement. As contact pressure increases, the surrounding tissue compresses, leading to increased attenuation of the arterial pressure signal. This compression can dampen the amplitude of the pulse wave due to tissue resistance, potentially impacting signal fidelity. Conversely, lower pressures, although more comfortable, risk suboptimal transducer contact, potentially leading to incomplete signal capture or susceptibility to noise.

Moreover, each subject’s unique tissue characteristics—including skin elasticity, subcutaneous fat, and vascular architecture—introduce variability in how the pulse wave attenuates through the tissue. By systematically varying contact pressure across this practical range, we were able to characterize the device’s ability to maintain accurate BP measurements across different levels of tissue compression and mechanical dampening. This approach provides critical insights into the device’s robustness and adaptability, ensuring that it can deliver reliable results across diverse user profiles and physiological conditions.

Testing commenced by activating the Finapres Nova and calibrating system pressure to ensure that the reconstructed brachial pressure aligned accurately with the upper arm cuff. Specifically, brachial cuff calibration was performed twice at the start of each data collection trial through the Finapres system. By subject, the systolic values ranged from 104.8 ± 2.6 mmHg to 173.2 ± 11.5 mmHg during brachial cuff calibration, and the diastolic values ranged from 61.3 ± 1.4 mmHg to 83.5 ± 8.2 mmHg. Before each static contact pressure testing trial, the Finapres was recalibrated to maintain measurement accuracy. Once calibration was complete, data collection for the device began, with the subject maintaining a static position to minimize motion artifacts. Synchronization between the devices was achieved by tapping the subject’s hand at the start of each session. Data was collected in three trials per contact pressure setting, each lasting one minute, resulting in nine minutes of static data. The subject remained still throughout testing to reduce variability and enhance signal clarity. Each subject completed 9 min of static data collection (3 min for 3 contact pressures).

#### 2.6.3. Data Collection/Dynamic Testing

After static testing, dynamic testing was conducted to assess the device’s performance under conditions that induce physiological changes in BP. The change in BP was induced using a leg press machine, where the subject’s BP was increased by varying the extension levels of the leg press. The device’s contact pressure was maintained at 40 mmHg for all dynamic trials, as this setting consistently provided the most accurate signal and demonstrated the most ideal attenuation coefficient during static testing based on our statistical analysis. We also selected this pressure to avoid higher contact ranges that could cause discomfort, ensuring signal fidelity and user comfort throughout the dynamic testing phase.

The dynamic testing aimed to evaluate how the device performs in conditions where BP fluctuates due to physical movement and exertion. The subject adjusted the weight on the leg press to a comfortable level that induced measurable BP changes. They extended their legs to three angles (150°, 120°, and 90°), expecting that more acute angles would induce a higher BP. Each subject completed five positions, including feet on the floor, feet on the leg press pad, and the three extended angles, with each position held for one minute. Each subject completed 15 min of dynamic data collection (5 min for 3 trials).

This setup allowed us to assess how the device responded to controlled variations in BP, simulating real-world physical activity. The device’s measurements were compared to the Finapres Nova’s to evaluate accuracy across a dynamic BP range.

Testing began with calibrating the Finapres Nova to ensure stable BP readings. Before each dynamic trial, the Finapres was recalibrated to maintain measurement consistency. Synchronization between the devices was achieved by tapping the subject’s hand at the start of each session. Data was collected over five minutes, with one minute allocated to each leg press position. The variation in leg angles provided a robust dataset for evaluating the device’s performance under dynamic physiological changes, with the Finapres Nova serving as the standard reference.

This comprehensive evaluation, combining both static and dynamic testing, allowed us to thoroughly characterize the device’s accuracy and responsiveness across a range of physiological states. The study provided critical insights into the device’s performance by considering factors such as tissue attenuation and mechanical dampening.

### 2.7. Data Processing

#### 2.7.1. System Characterization

Static data processing was performed in MATLAB to evaluate the effect of contact pressure on waveform attenuation, with measurements collected at controlled pressures of 20, 30, and 40 mmHg. Three trials were conducted per pressure level for each subject to ensure reliability. Attenuation coefficients (α) were derived using Equation (2) by comparing changes in the systolic peak amplitude of the device’s arterial waveform to those measured concurrently by the Finapres, which served as the reference standard.

The model fit was applied to the experimental data to characterize the relationship between attenuation and contact pressure. Mean attenuation coefficients and standard deviations were computed across trials for each pressure level. This model-driven approach informed compensatory strategies for waveform morphology adjustments during real-time BP monitoring, ensuring accurate signal reconstruction under varying contact pressures.

The attenuation of the arterial pressure signal as it propagates through tissue is a key factor in accurate blood pressure monitoring. In this study, we assessed the effect of static contact pressure on the attenuation coefficient (α) to better understand its role in the signal transmission pathway. The attenuation coefficient was derived using the following equation:

(8) $$P_{measured}(t)=P_{arterial}\left(t\right)*e^{-\alpha d}$$

where P_measured_ is the pressure detected by the sensor, P_arterial_ is the true arterial pressure, α is the attenuation coefficient, and d represents the effective depth of the artery beneath the skin. While d is a known physiological parameter, it was not directly measured in this study. Instead, its effects are inherently captured within the attenuation coefficient α, which accounts for tissue properties, arterial depth, and pressure transmission.

As contact pressure increases, tissue compression reduces d, altering the effective depth at which the signal propagates. However, rather than requiring a direct measurement of d, the calculated α effectively compensates for these variations, ensuring an accurate BP estimation.

#### 2.7.2. Dynamic Testing

**Figure 4:**
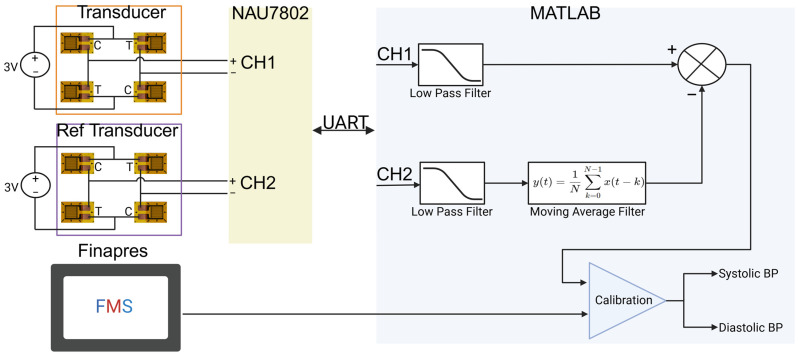
Block diagram of the signal acquisition, processing, and calibration pipeline. The system uses interchangeable primary and reference transducers, each powered by a 3 V source and configured in a Wheatstone bridge to capture arterial pressure signals. Outputs (CH1 and CH2) are digitized via an NAU7802 ADC and transmitted to MATLAB through a UART interface. In MATLAB, both channels are low-pass filtered to remove high-frequency noise. The reference signal undergoes additional smoothing with a moving average filter and is subtracted from the primary signal to reduce common-mode noise and isolate the arterial pulse waveform. The resulting calibrated output is mapped to systolic and diastolic BP values, using baseline measurements from a Finapres Medical System (FMS) for calibration.

This study implemented dynamic data processing in MATLAB to address noise artifacts and real-time contact pressure variations, with data streamed live via UART, as shown in Figure 4. First, a low-pass filter was applied to both the artery and reference signals to attenuate high-frequency noise generated by minor movements. Next, the reference signal was subtracted from the artery signal after smoothing with a moving average, effectively reducing the baseline drift caused by gradual contact pressure changes. Calibration was then performed by comparing the processed arterial waveform to baseline waveforms obtained during Finapres-assisted measurements, allowing for morphology adjustments to ensure accuracy. Finally, to validate the effectiveness of the filtering process, small, controlled movements were introduced to observe the signal’s stability, with filter parameters adjusted as necessary to optimize performance under dynamic conditions. This approach enabled continuous and reliable cuffless BP monitoring with minimized noise and compensation for contact pressure variability.

**Figure 5:**
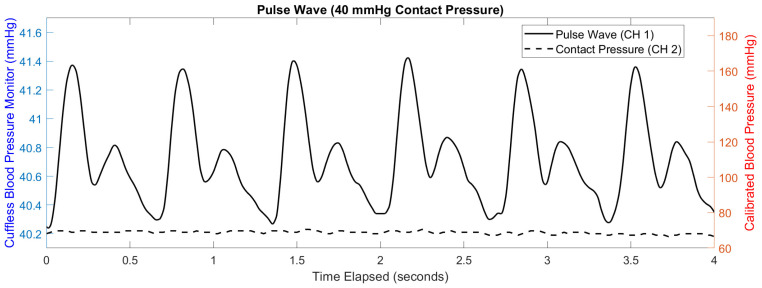
Sample segment of raw pressure-patch data and calibrated blood pressure values obtained from Finapres reference measurements. The left y-axis shows the strain gauge waveform, which reflects the contact pressure combined with the arterial pulse signal detected by the sensor. The right y-axis shows the calibrated blood pressure in mmHg. This signal represents live blood pressure values plotted over time, derived from the channel 1 transducer after low-pass filtering and calibration.

To demonstrate the signal characteristics after filtering, a representative segment of the processed waveform is shown in Figure 5. This pulse wave was acquired from the primary transducer following low-pass filtering and is time-aligned with corresponding blood pressure values obtained from the Finapres reference system. The figure illustrates the distinct morphological features of the arterial pressure signal captured by the sensor, validating the system’s ability to resolve individual pulse cycles during dynamic testing.

## 3. Results

### 3.1. Static Contact Pressure Analysis and Attenuation Assessment

We applied the attenuation coefficient model described in Section 2.7.1 to assess how α varied across different static contact pressures. Static pressures of 20, 30, 40, 50, and 60 mmHg were applied sequentially. For each level, three trials were conducted across 10 subjects. Attenuation coefficients were calculated using Equation (9):

(9) $$\alpha =-\ln(\frac{P_{measured}}{P_{arterial}})$$

To ensure consistent reference pressure values P_arterial_, all measurements were calibrated against Finapres-derived arterial pressure values under static conditions. While the Finapres NOVA is not a gold standard like invasive arterial catheterization, nor as common clinically as cuff-based BP measurements, it is widely used in research for beat-to-beat BP monitoring and offers real-time BP tracking, making it a more appropriate reference for evaluating our wearable device designed for continuous monitoring.

Since contact pressure affects tissue compression, and thereby effective arterial depth, α inherently reflects variations in tissue thickness and impedance. This approach yields a pressure-dependent attenuation coefficient that accounts for anatomical variability. The analysis revealed that α decreased linearly with increasing contact pressure. The calculated α values were as follows:

- At 20 mmHg: α_20_ = 5.0 ± 0.1;
- At 30 mmHg: α_30_ = 4.8 ± 0.2;
- At 40 mmHg: α_40_ = 4.5 ± 0.1;
- At 50 mmHg: α_50_ = 4.2 ± 0.1;
- At 60 mmHg: α_60_ = 3.9 ± 0.1;

where X and Y represent the mean and standard deviation of α across subjects, respectively.

The attenuation coefficient decreased uniformly between each contact pressure increment, indicating that for a reasonable range of contact pressures, the attenuation behavior of the underlying tissues is linearly decreasing. This behavior aligns with theoretical expectations, as increased contact pressure improves transmission up to a limit dictated by tissue mechanics.

### 3.2. Model-Based Analysis

To further interpret the attenuation trend, we modeled α as a linear function of contact pressure P_c_:

(10) α=m∗Pc+b

where m is the slope, and b is the intercept. This model effectively captures the observed linear decrease in α with an increasing P_c_, consistent with the physical interpretation that improved coupling efficiency reduces signal attenuation as contact pressure increases. However, in biological tissues, attenuation decreases with increasing contact pressure until structural changes occur, such as arterial occlusion.

The fitted parameters derived from the experimental data were

*m* = −0.028

*b* = 5.56

**Figure 6:**
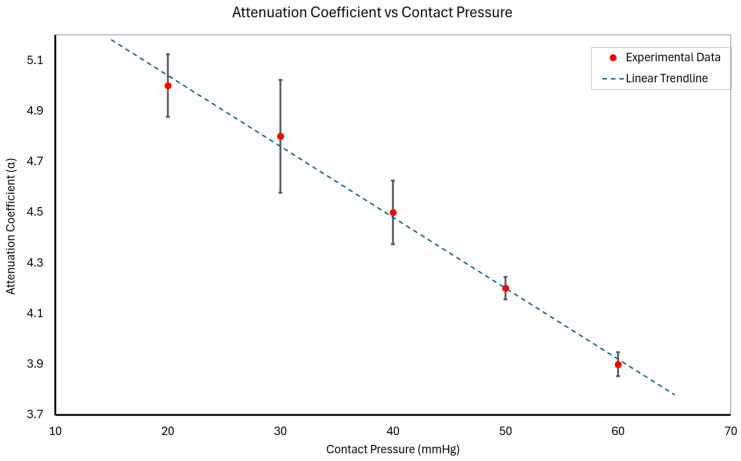
Relationship between attenuation coefficient (α) and contact pressure. The experimental data (red points) illustrate the effect of varying contact pressures on the attenuation coefficient, with error bars representing the standard deviation. The dotted blue line represents the model fit, showcasing the predictive capability of the proposed model under varying conditions.

The model, shown in Figure 6 above, fits the experimental data well, with a slope of m = −0.028 and an intercept of b = 5.56. This supports the theory that increased pressure enhances mechanical coupling, reducing attenuation, up to the point of diminishing returns or structural limitations like arterial occlusion.

This model offers a framework for refining calibration strategies and adapting the system to individual tissue profiles.

### 3.3. Assessment of Device Performance Under Dynamic Physiological Conditions

**Figure 7:**
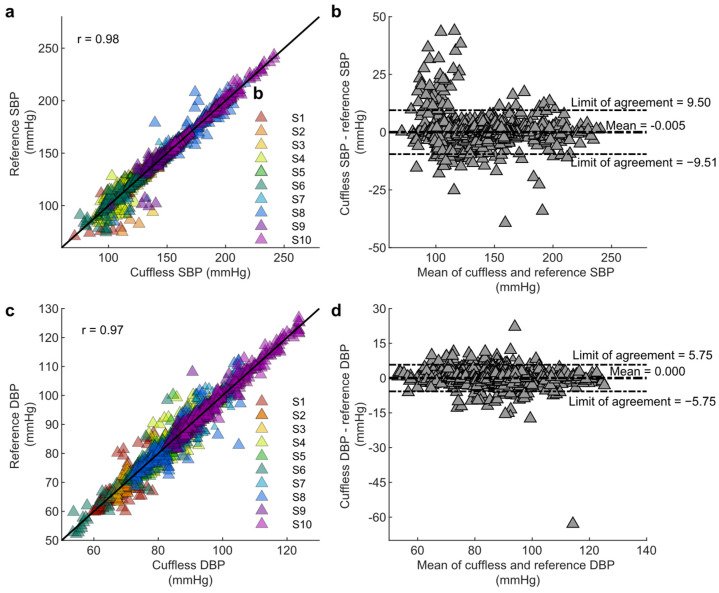
Validation of cuffless blood pressure measurements compared to reference values across multiple subjects. (**a**) Correlation plot of systolic blood pressure (SBP) measurements from the cuffless device versus reference SBP values. (**b**) Bland–Altman plot for SBP, illustrating the agreement between cuffless and reference SBP measurements. (**c**) Correlation plot of diastolic blood pressure (DBP) measurements from the cuffless device versus reference DBP values. (**d**) Bland–Altman plot for DBP, illustrating the agreement between cuffless and reference DBP measurements.

The device’s accuracy was evaluated under dynamic BP conditions using a leg press protocol designed to induce controlled blood pressure changes. Figure 7 illustrates the strong agreement between the wearable device and the Finapres reference.

Pearson correlation coefficients of r = 0.98 for systolic BP and r = 0.97 for diastolic BP were observed, both statistically significant (*p* < 0.001). A Bland–Altman analysis confirmed negligible bias, with a mean bias of 0.005 mmHg for systolic BP and 0 mmHg for diastolic BP, and standard deviations of 3.87 mmHg and 2.57 mmHg, respectively. The 95% limits of agreement (LoAs) ranged from −9.51 to 9.50 mmHg for systolic and −5.75 to 5.75 mmHg for diastolic, aligning with clinically acceptable standards.

The device achieved a MAE of 2.45 ± 3.99 mmHg for systolic BP and 1.59 ± 2.08 mmHg for diastolic BP, meeting accuracy requirements for clinical relevance. Some deviations in the systolic range of 90–120 mmHg appeared in a few subjects, likely due to transient spikes in the Finapres signal during movement. These anomalies highlight the potential for improved robustness in the strain gauge-based device under dynamic conditions.

### 3.4. Comparison of Device Accuracy with Reference Standard

**Figure 8:**
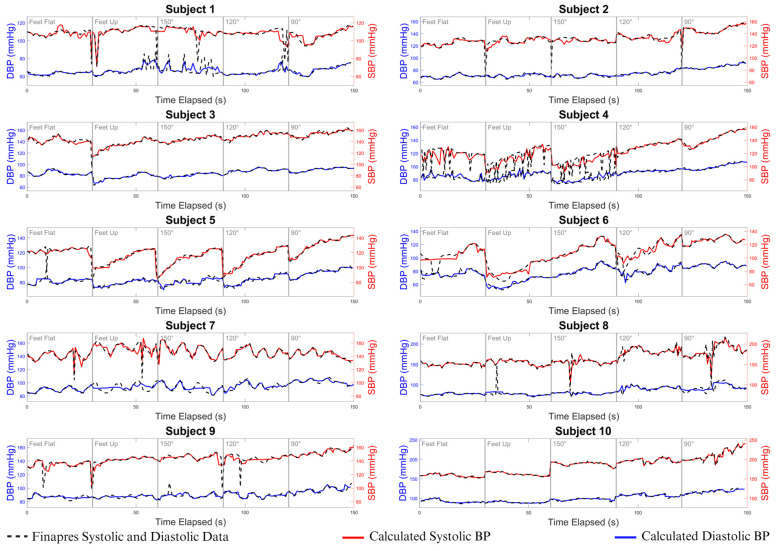
Comparison of cuffless blood pressure estimations with Finapres measurements during dynamic testing. The 150 s data segment shown represents a single dynamic testing trial, with 30 s increments extracted from the full dataset for each position. Beat-to-beat BP values were continuously recorded and compared between our device and Finapres to ensure accurate dynamic BP tracking. SBP (red line) and DBP (blue line) estimations with the cuffless blood pressure wearable device, compared to standard Finapres finger BP cuff measurements (black dotted line) for each participant.

Figure 8 presents time-aligned systolic and diastolic BP measurements from both the wearable device and the Finapres reference across multiple subjects during dynamic testing.

The device’s BP estimations track closely with the Finapres signals, demonstrating consistent performance under varying physiological conditions. While the overall agreement remained strong, some subjects (e.g., 1 and 4) exhibited transient deviations in the Finapres signal that were not mirrored by the wearable device. These artifacts may reflect movement sensitivity in the Finapres system rather than inaccuracies in the strain gauge-based device.

The close alignment in both magnitude and trend further supports the device’s ability to capture continuous BP dynamics with clinical accuracy, reinforcing the findings from Section 3.3.

## 4. Discussion

### 4.1. Comparison to Prior Work and State-of-the-Art Devices

Continuous BP monitoring is vital for diagnosing and managing hypertension effectively. However, existing cuffless solutions, such as ultrasound and BIOZ-based devices, suffer from significant drawbacks, including bulky hardware, frequent recalibration, and susceptibility to poor arterial contact, leading to noise and a degraded signal quality. Other emerging technologies, such as PPG, epidermal strain sensors, radar, microwave, and acoustic sensors, face limitations related to skin tone sensitivity, high costs, complex algorithms, and increased power demands, further restricting their practicality for widespread, long-term use.

**Figure 9:**
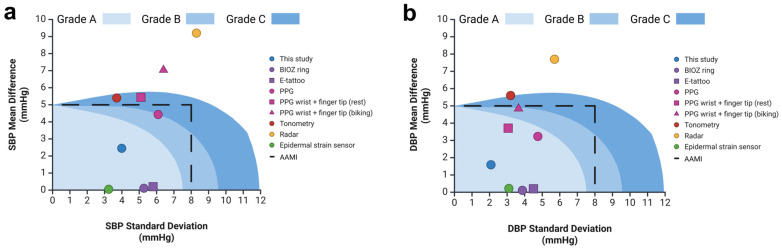
Comparative analysis of cuffless blood pressure estimation methods: Performance comparison of this work and related studies for cuffless systolic blood pressure (SBP, (**a**)) and diastolic blood pressure (DBP, (**b**)) estimation. The shaded regions, light blue to dark blue, denote Grade A, Grade B, and Grade C classifications as defined by the BHS standard. Data points within each region indicate the classification achieved by various methods, including BIOZ rings, e-tattoos, tonometry, radar, and PPG-based approaches. The dashed black line represents the error limits defined by the AAMI standard, demarcating acceptable levels of accuracy.

In contrast, as shown in Figure 9, our strain gauge-based wearable device overcomes these challenges. Additional data and figures are provided in the Supplementary Materials. It eliminates the need for external recalibration, significantly lowers power consumption, lowers cost, and achieves BP accuracy comparable to conventional cuff-based sphygmomanometers. The simplified design and reduced reliance on complex algorithms enhance the feasibility of continuous, cuffless BP monitoring in a low-cost, user-friendly form factor. This combination of accuracy, comfort, and efficiency positions our device as a more practical solution for long-term hypertension management than existing technologies.

### 4.2. Limitations

While the device demonstrated promising accuracy for continuous BP monitoring, several limitations must be considered in this study. First, the Finapres NOVA, which was used as the reference standard, relies on the vascular unloading technique. This method can introduce inaccuracies in continuous BP measurements, particularly during dynamic conditions where physiological changes occur rapidly. The vascular unloading technique adjusts pressure in the finger cuff to maintain a constant blood volume, but this process can result in baseline shifts, which complicates direct comparisons with our device. These shifts are known to distort BP readings, especially during movements that alter peripheral circulation, such as force exertion in leg press exercises [57]. In such scenarios, the Finapres may overestimate systolic BP and underestimate diastolic BP, contributing to the variability in its accuracy, particularly when central and peripheral pressures diverge under physical stress [58].

In addition to these technical limitations, the study sample was relatively small and homogeneous, consisting primarily of healthy individuals with few comorbidities, and the device’s performance across diverse populations with hypertension must be more thoroughly evaluated. Further, although the device is agnostic to skin tone, other parameters such as vasculature, fat percentage, and body composition variations must be accounted for to ensure measurement accuracy. Otherwise, this limits the generalizability of the findings, as the performance of both the Finapres and the device may vary in populations with different cardiovascular conditions or health profiles. Individuals with peripheral vascular issues, for instance, may experience greater discrepancies between central and peripheral BP measurements, further complicating the use of the Finapres as a reliable standard in such populations [59]. In addition, while the Finapres NOVA served as a valuable reference for continuous BP measurement in this study, its limitations should be acknowledged, particularly the potential for baseline shifts and inaccuracies during physical exertion. Furthermore, although varying leg press extension levels allowed for a good dynamic range for BP, future studies should evaluate the device across a broader spectrum of activities, such as running, cycling, or resistance exercises, to better understand how it performs in real-world, dynamic conditions.

## 5. Conclusions

This study demonstrates the development and validation of a novel strain gauge technology for continuous, accurate, reusable, and noninvasive cuffless blood pressure (BP) monitoring. The wearable device achieves an accuracy comparable to traditional cuff-based systems while addressing key limitations such as user discomfort, intermittent readings, and the reliance on disposables. Its compact, cost-effective design enables real-time BP monitoring with minimal user interference, supporting long-term use.

The dual strain gauge setup improves accuracy and usability by allowing either transducer to be placed on or near the artery, reducing alignment challenges across varied anatomies. The reusable design also reduces cost and waste, promoting sustainability. This reusable design uses adhesive materials, minimizing the risk of skin irritation. The sensors typically will not exceed 60 mmHg, which will minimize skin indentation and irritation.

The modeled relationship between attenuation coefficient α and contact pressure Pc confirms the system’s robustness, with a linear trend supporting calibration refinement. Variability between measured and modeled values highlights the impact of anatomical differences, which future work will address to improve its generalizability.

The strong correlation with reference measurements (r = 0.98 systolic, r = 0.97 diastolic) underscores the device’s clinical potential. Despite some variability due to the reference method, the results remained within clinically acceptable accuracy limits.

While these results are promising, this work represents an early-stage validation step. Further studies involving broader and more diverse populations, extended wear trials, and performance comparisons in dynamic, real-world environments will be essential. Clinical trials and regulatory pathways must also be pursued before widespread adoption can be realized.

In summary, these findings position the device as a transformative, low-cost, accessible solution for managing hypertension. Future iterations will address the current limitations, enabling seamless integration into daily life and advancing the early detection of cardiovascular conditions to reduce the global burden of CVD.

## 6. Patents

Two patents have been generated with the development of the work for this publication: WO2025076198—Multimodal Sensing Patch with Active Contact Pressure Control and Quantification for Physiological Measurements and Biomarker Detection, and US20240225546A1—Integrated Pressure Transducer for Precise Quantification of Applied Surface Force in Wearable Devices.

## Figures and Tables

**Table 1:** Comparison of strain gauge-based blood pressure monitoring with PPG, BioZ, and acoustic technologies.

Feature	Strain Gauge-Based Device	PPG-Based Devices	BioZ-Based Devices	Acoustic Devices
Accuracy and Sensitivity	High	Low [40]	Moderate [41]	Low [42]
Impact of Skin Tone	None	Yes [20]	None *	None *
Influence of BMI	Moderate	High [20]	Moderate [43]	Moderate [44]
Motion Artifact Sensitivity	Moderate	High [40]	Moderate [45]	High [42]
Suitability for Continuous Monitoring	High	Moderate [20]	Moderate [29]	Moderate [46]
Need for Recalibration	No	Yes [47]	Yes [48]	Yes [49]

* To our knowledge, no studies have examined this parameter; such an influence is unlikely.

## Data Availability

Data will be made available on request.

## Supplementary Materials

The following supporting information can be downloaded at: https://www.mdpi.com/article/10.3390/bios15070413/s1. Table S1. *Comparative Analysis of Cuffless Blood Pressure Estimation Methods.* This table summarizes the numerical information represented in Supplementary Figure 1. AAMI pass/fail status is listed for each device or method. The numerical mean error and standard deviation values for the BIOZ ring [29], bioimpedance e-tattoo [48], single-point PPG [60], and epidermal strain sensor [51] meet AAMI standards, while the wrist-to-fingertip PPG (stationary and biking) [50], tonometry [61], and radar-based methods [26] do not. Table S2. *Summary of Physiological Interventions and Corresponding Effects on Blood Pressure.* Summarizes the procedures and outcomes (ΔSBP and ΔDBP) from published literature for cold pressor, sustained handgrip, and Valsalva interventions. Table S3. *Ranges in Systolic and Diastolic Blood Pressure from Baseline Across 10 Participants.* Reports the absolute deviation from baseline (Δ = (max − min)/2) for systolic and diastolic BP in each participant. Table S4. *Full Systolic and Diastolic Blood Pressure Ranges per Subject.* Presents full min-to-max BP ranges recorded for each subject during all test conditions. References [62,63,64,65,66,67,68] are cited in the supplementary materials

## Abbreviations

ADC	Analog-to-Digital Converter
AC	Alternating Current
AAMI	Association for the Advancement of Medical Instrumentation
BMI	Body Mass Index
BP	Blood Pressure
BHS	British Hypertension Society
BIOZ	Bioimpedance
CVD	Cardiovascular Disease
DBP	Diastolic Blood Pressure
DC	Direct Current
FMS	Finapres Medical System
I2C	Inter-Integrated Circuit
IRB	Institutional Review Board
LoA	Limits of Agreement
MAE	Mean Absolute Error
MmHg	Millimeters of Mercury
NOVA	Noninvasive Vascular Analyzer
PPG	Photoplethysmography
PTT	Pulse Transit Time
Pc	Contact Pressure
SBP	Systolic Blood Pressure
UART	Universal Asynchronous Receiver/Transmitter
Z	Mechanical Impedance

## References

[1] Di Cesare M., Perel P., Taylor S., Kabudula C., Bixby H., Gaziano T.A., McGhie D.V., Mwangi J., Pervan B., Narula J. (2024). The heart of the world. Glob. Heart.

[2] Tackling G., Borhade M.B. (2023). Hypertensive heart disease. StatPearls.

[3] Powell-Wiley T.M., Baumer Y., Baah F.O., Baez A.S., Farmer N., Mahlobo C.T., Pita M.A., Potharaju K.A., Tamura K., Wallen G.R. (2022). Social determinants of cardiovascular disease. Circ. Res..

[4] Charchar F.J., Prestes P.R., Mills C., Ching S.M., Neupane D., Marques F.Z., Sharman J.E., Vogt L., Burrell L.M., Korostovtseva L. (2024). Lifestyle management of hypertension: International Society of Hypertension position paper endorsed by the World Hypertension League and European Society of Hypertension. J. Hypertens..

[5] Ogedegbe G., Pickering T. (2010). Principles and techniques of blood pressure measurement. Cardiol. Clin..

[6] Nitzan M., Slotki I., Shavit L. (2017). More accurate systolic blood pressure measurement is required for improved hypertension management: A perspective. Med. Devices Evid. Res..

[7] Mirdamadi A., Etebari M. (2017). Comparison of manual versus automated blood pressure measurement in intensive care unit, coronary care unit, and emergency room. ARYA Atheroscler..

[8] Khan Mamun M.M.R., Sherif A. (2022). Advancement in the cuffless and noninvasive measurement of blood pressure: A review of the literature and open challenges. Bioengineering.

[9] Bilo G., Sala O., Perego C., Faini A., Gao L., Głuszewska A., Ochoa J.E., Pellegrini D., Lonati L.M., Parati G. (2017). Impact of cuff positioning on blood pressure measurement accuracy: May a specially designed cuff make a difference?. Hypertens. Res..

[10] Mahe G., Comets E., Nouni A., Paillard F., Dourmap C., Le Faucheur A., Jaquinandi V. (2017). A minimal resting time of 25 min is needed before measuring stabilized blood pressure in subjects addressed for vascular investigations. Sci. Rep..

[11] Zheng D., Giovannini R., Murray A. (2012). Effect of respiration, talking and small body movements on blood pressure measurement. J. Hum. Hypertens..

[12] Pioli M.R., Ritter A.M., de Faria A.P., Modolo R. (2018). White coat syndrome and its variations: Differences and clinical impact. Integr. Blood Press. Control.

[13] Eguchi K., Kuruvilla S., Ogedegbe G., Gerin W., Schwartz J.E., Pickering T.G. (2009). What is the optimal interval between successive home blood pressure readings using an automated oscillometric device?. J. Hypertens..

[14] Sharma M., Barbosa K., Ho V., Griggs D., Ghirmai T., Krishnan S.K., Hsiai T.K., Chiao J.-C., Cao H. (2017). Cuff-less and continuous blood pressure monitoring: A methodological review. Technologies.

[15] Konstantinidis D., Iliakis P., Tatakis F., Thomopoulos K., Dimitriadis K., Tousoulis D., Tsioufis K. (2022). Wearable blood pressure measurement devices and new approaches in hypertension management: The digital era. J. Hum. Hypertens..

[16] Charlton P.H., Allen J., Bailón R., Baker S., Behar J.A., Chen F., Clifford G.D., Clifton D.A., Davies H.J., Ding C. (2023). The 2023 wearable photoplethysmography roadmap. Physiol. Meas..

[17] Elgendi M., Fletcher R., Liang Y., Howard N., Lovell N.H., Abbott D., Lim K., Ward R. (2019). The use of photoplethysmography for assessing hypertension. npj Digit. Med..

[18] Schutte A.E., Kollias A., Stergiou G.S. (2022). Blood pressure and its variability: Classic and novel measurement techniques. Nat. Rev. Cardiol..

[19] Scardulla F., Cosoli G., Spinsante S., Poli A., Iadarola G., Pernice R., Busacca A., Pasta S., Scalise L., D’Acquisto L. (2023). Photoplethysmograhic sensors, potential and limitations: Is it time for regulation? A comprehensive review. Measurement.

[20] Fine J., Branan K.L., Rodriguez A.J., Boonya-Ananta T., Ramella-Roman J.C., McShane M.J., Cote G.L. (2021). Sources of inaccuracy in photoplethysmography for continuous cardiovascular monitoring. Biosensors.

[21] Rodriguez A.J., Boonya-Ananta M.T., Gonzalez M., Le V.N.D., Fine J., Palacios C., McShane M.J., Coté G.L., Ramella-Roman J.C. (2022). Skin optical properties in the obese and their relation to body mass index: A review. J. Biomed. Opt..

[22] Moraes J.L., Rocha M.X., Vasconcelos G.G., Vasconcelos Filho J.E., De Albuquerque V.H.C., Alexandria A.R. (2018). Advances in photopletysmography signal analysis for biomedical applications. Sensors.

[23] Finnegan E., Davidson S., Harford M., Watkinson P., Tarassenko L., Villarroel M. (2023). Features from the photoplethysmogram and the electrocardiogram for estimating changes in blood pressure. Sci. Rep..

[24] El-Hajj C., Kyriacou P.A. (2020). A review of machine learning techniques in photoplethysmography for the non-invasive cuff-less measurement of blood pressure. Biomed. Signal Process. Control.

[25] Ibrahim B., Jafari R. (2019). Cuffless blood pressure monitoring from an array of wrist bio-impedance sensors using subject-specific regression models: Proof of concept. IEEE Trans. Biomed. Circuits Syst..

[26] Vysotskaya N., Will C., Servadei L., Maul N., Mandl C., Nau M., Harnisch J., Maier A. (2023). Continuous non-invasive blood pressure measurement using 60 GHz-radar—A feasibility study. Sensors.

[27] Suzuki S., Sun G., Hoshiga M., Kotani K., Asao T. (2022). Noncontact Monitoring of Relative Changes in Blood Pressure Using Microwave Radar Sensors. J. Biomed. Sci. Eng..

[28] Sharma P., Imtiaz S.A., Rodriguez-Villegas E. (2019). Acoustic sensing as a novel wearable approach for cardiac monitoring at the wrist. Sci. Rep..

[29] Sel K., Osman D., Huerta N., Edgar A., Pettigrew R.I., Jafari R. (2023). Continuous cuffless blood pressure monitoring with a wearable ring bioimpedance device. npj Digit. Med..

[30] Naranjo-Hernández D., Reina-Tosina J., Min M. (2019). Fundamentals, recent advances, and future challenges in bioimpedance devices for healthcare applications. J. Sens..

[31] Goyal K., Borkholder D.A., Day S.W. (2022). Dependence of skin-electrode contact impedance on material and skin hydration. Sensors.

[32] Ibrahim B., McMurray J., Jafari R. (2018). A wrist-worn strap with an array of electrodes for robust physiological sensing. Proceedings of the 2018 40th Annual International Conference of the IEEE Engineering in Medicine and Biology Society (EMBC).

[33] Alvarez J.T., Gerez L.F., Araromi O.A., Hunter J.G., Choe D.K., Payne C.J., Wood R.J., Walsh C.J. (2022). Towards soft wearable strain sensors for muscle activity monitoring. IEEE Trans. Neural Syst. Rehabil. Eng..

[34] Wu H., Yang G., Zhu K., Liu S., Guo W., Jiang Z., Li Z. (2021). Materials, devices, and systems of on-skin electrodes for electrophysiological monitoring and human–machine interfaces. Adv. Sci..

[35] Viana G., Costa M., Banea M., Da Silva L. (2017). A review on the temperature and moisture degradation of adhesive joints. Proc. Inst. Mech. Eng. Part L J. Mater. Des. Appl..

[36] Lai H., Liu Y., Cheng Y., Shi L., Wang R., Sun J. (2023). Temperature-Triggered Adhesive Bioelectric Electrodes with Long-Term Dynamic Stability and Reusability. Adv. Sci..

[37] Kummerow C.D., Poczatek J.C., Almond S., Berg W., Jarrett O., Jones A., Kantner M., Kuo C.-P. (2021). Hyperspectral microwave sensors—Advantages and limitations. IEEE J. Sel. Top. Appl. Earth Obs. Remote Sens..

[38] Alahnomi R.A., Zakaria Z., Yussof Z.M., Althuwayb A.A., Alhegazi A., Alsariera H., Rahman N.A. (2021). Review of recent microwave planar resonator-based sensors: Techniques of complex permittivity extraction, applications, open challenges and future research directions. Sensors.

[39] Islam S.M.M. (2022). Radar-based remote physiological sensing: Progress, challenges, and opportunities. Front. Physiol..

[40] Mehta S., Kwatra N., Jain M., McDuff D. (2024). Examining the challenges of blood pressure estimation via photoplethysmogram. Sci. Rep..

[41] Xu J., Harpe P., Van Hoof C. (2018). An energy-efficient and reconfigurable sensor IC for bio-impedance spectroscopy and ECG recording. IEEE J. Emerg. Sel. Top. Circuits Syst..

[42] Kong F., Zou Y., Li Z., Deng Y. (2024). Advances in portable and wearable acoustic sensing devices for human health monitoring. Sensors.

[43] Brown C.V., Martin M.J., Shoemaker W.C., Wo C.C., Chan L., Azarow K., Demetriades D. (2005). The effect of obesity on bioimpedance cardiac index. Am. J. Surg..

[44] Li J., Jia H., Zhou J., Huang X., Xu L., Jia S., Gao Z., Yao K., Li D., Zhang B. (2023). Thin, soft, wearable system for continuous wireless monitoring of artery blood pressure. Nat. Commun..

[45] Van Helleputte N., Konijnenburg M., Pettine J., Jee D.-W., Kim H., Morgado A., Van Wegberg R., Torfs T., Mohan R., Breeschoten A. (2014). A 345 µW multi-sensor biomedical SoC with bio-impedance, 3-channel ECG, motion artifact reduction, and integrated DSP. IEEE J. Solid-State Circuits.

[46] Wang C., Li X., Hu H., Zhang L., Huang Z., Lin M., Zhang Z., Yin Z., Huang B., Gong H. (2018). Monitoring of the central blood pressure waveform via a conformal ultrasonic device. Nat. Biomed. Eng..

[47] Wang G., Atef M., Lian Y. (2018). Towards a continuous non-invasive cuffless blood pressure monitoring system using PPG: Systems and circuits review. IEEE Circuits Syst. Mag..

[48] Kireev D., Sel K., Ibrahim B., Kumar N., Akbari A., Jafari R., Akinwande D. (2022). Continuous cuffless monitoring of arterial blood pressure via graphene bioimpedance tattoos. Nat. Nanotechnol..

[49] Mercier L., Langø T., Lindseth F., Collins D.L. (2005). A review of calibration techniques for freehand 3-D ultrasound systems. Ultrasound Med. Biol..

[50] Wang Y.-J., Chen C.-H., Sue C.-Y., Lu W.-H., Chiou Y.-H. (2018). Estimation of blood pressure in the radial artery using strain-based pulse wave and photoplethysmography sensors. Micromachines.

[51] Li S., Wang H., Ma W., Qiu L., Xia K., Zhang Y., Lu H., Zhu M., Liang X., Wu X.-E. (2023). Monitoring blood pressure and cardiac function without positioning via a deep learning–assisted strain sensor array. Sci. Adv..

[52] Hu J.-R., Martin G., Iyengar S., Kovell L.C., Plante T.B., van Helmond N., Dart R.A., Brady T.M., Turkson-Ocran R.-A.N., Juraschek S.P. (2023). Validating cuffless continuous blood pressure monitoring devices. Cardiovasc. Digit. Health J..

[53] de Sousa N.M., Magosso R.F., Dipp T., Plentz R.D., Marson R.A., Montagnolli A.N., Martins R.A., Perez S.E., Baldissera V. (2014). Continuous blood pressure response at different intensities in leg press exercise. Eur. J. Prev. Cardiol..

[54] Gallagher D., Heymsfield S.B., Heo M., Jebb S.A., Murgatroyd P.R., Sakamoto Y. (2000). Healthy percentage body fat ranges: An approach for developing guidelines based on body mass index. Am. J. Clin. Nutr..

[55] Wang L., Tian S., Zhu R. (2023). A new method of continuous blood pressure monitoring using multichannel sensing signals on the wrist. Microsyst. Nanoeng..

[56] Scardulla F., D’Acquisto L., Colombarini R., Hu S., Pasta S., Bellavia D. (2020). A study on the effect of contact pressure during physical activity on photoplethysmographic heart rate measurements. Sensors.

[57] Imholz B.P., Montfrans G.A.V., Settels J.J., Hoeven G.M.V.D., Karemaker J.M., Wieling W. (1988). Continuous non-invasive blood pressure monitoring: Reliability of Finapres device during the Valsalva manoeuvre. Cardiovasc. Res..

[58] Silke B., McAuley D. (1998). Accuracy and precision of blood pressure determination with the Finapres: An overview using re-sampling statistics. J. Hum. Hypertens..

[59] Ismail S.N.A., Nayan N.A., Jaafar R., May Z. (2022). Recent advances in non-invasive blood pressure monitoring and prediction using a machine learning approach. Sensors.

[60] Yang S., Zhang Y., Cho S.-Y., Correia R., Morgan S.P. (2021). Non-invasive cuff-less blood pressure estimation using a hybrid deep learning model. Opt. Quantum Electron..

[61] Kemmotsu O., Ueda M., Otsuka H., Yamamura T., Okamura A., Ishikawa T., Winter D.C., Eckerle J.S. (1991). Blood Pressure Measurement by Arterial Tonometry in Controlled Hypotension. Anesthesia Analg..

[62] Hines E.A., Brown G.E. (1936). The cold pressor test for measuring the reactibility of the blood pressure: Data concerning 571 normal and hypertensive subjects. Am. Hear. J..

[63] Hanson P., Nagle F. (1987). Isometric Exercise: Cardiovascular Responses in Normal and Cardiac Populations. Cardiol. Clin..

[64] Petrofsky J.S., Lind A.R. (1975). Aging, isometric strength and endurance, and cardiovascular responses to static effort. J. Appl. Physiol..

[65] Martin C.E., Shaver J.A., Leon D.F., Thompson M.E., Reddy P.S., Leonard J.J. (1974). Autonomic Mechanisms in Hemodynamic Responses to Isometric Exercise. J. Clin. Investig..

[66] Petrofsky J.S., Burse R.L., Lind A.R. (1975). Comparison of physiological responses of women and men to isometric exercise. J. Appl. Physiol..

[67] Parati G., Casadei R., Groppelli A., Di Rienzo M., Mancia G. (1989). Comparison of finger and intra-arterial blood pressure monitoring at rest and during laboratory testing. Hypertension.

[68] Mukkamala R., Hahn J.-O., Inan O.T., Mestha L.K., Kim C.-S., Toreyin H., Kyal S. (2015). Toward Ubiquitous Blood Pressure Monitoring via Pulse Transit Time: Theory and Practice. IEEE Trans. Biomed. Eng..

