# Supplementary material for: A Novel Wearable Device for Continuous Blood Pressure Monitoring Utilizing Strain Gauge Technology
Source: Biosensors (Basel). 2025 Jun 27;15(7):413. doi: 10.3390/bios15070413 (PMC12294096; doi:10.3390/bios15070413)
Supplement: Supplementary file 1 [file biosensors-15-00413-s001.zip › biosensors-3669612-supplementary.pdf]

Supplementary Information

Table S1: Comparative Analysis of Cuffless Blood Pressure Estimation Methods.

Device	Mode	AAMI*	Use	SBP Error (mean +/- std.) in mmHg	DBP Error (mean +/- std.) in mmHg
This study	Strain gauge based	Pass	At rest and leg press	2.45 +/- 3.99	1.59 +/- 2.08
BIOZ ring	BIOZ	Pass	At rest & cold pressor	0.11 +/- 5.27	0.11 +/- 3.87
E-tattoo	BIOZ	Pass	Hand press & cold press	0.2 +/- 5.8	0.2 +/- 4.5
PPG	PPG + ECG	Pass	At rest & running down then up 4 floors	4.43 +/- 6.09	3.23 +/- 4.75
PPG wrist + finger tip	PPG	Fail	At rest	5.44 +/- 5.10	3.71 +/- 3.06
PPG wrist + finger tip	PPG	Fail	Biking	7.04 +/- 6.40	4.84 +/- 3.65
Tonometry	Arterial Tonometry	Fail	Under anesthesia	5.4 +/- 3.7	5.6 +/- 3.2
Radar	Radar	Fail	At rest	9.2 +/- 8.3	7.7 +/- 5.7
Epidermal strain sensor	Epidermal strain	Pass	At rest	0.05 +/- 3.24	0.22 +/- 3.10

AAMI = Association for the Advancement of Medical Instrumentation
*To meet AAMI criteria the mean difference between the device and the mercury standard must be <5 mmHg or the standard deviation must be <8 mmHg

Supplementary Table 1 summarizes the numerical information represented in Supplemental Figure 1. An additional column details the AAMI status of each method or device. The numerical mean error and standard deviation values for the BIOZ ring [29], bioimpedance e-tattoo [48], single-point PPG [60], and epidermal strain sensor [51] pass according to the AAMI standards while the wrist to fingertip PPG while stationary and while biking [50], tonometry [61], and radar [26] fail according to these standards. The table includes additional details as to the study conditions under which each device or method was tested.

Table S2: Summary of Physiological Interventions and Corresponding Effects on Blood Pressure.

Intervention	Procedure	□SBP (mmHg)	□DBP (mmHg)
Cold Pressor (Hyporeactors) [48][62]	Hand in 4°C water for 1 minute	+11.4 (from normal)	+10.6 (from normal)
Cold Pressor (Hyperreactors) [29][62]	Hand in 4°C water for 1 minute	+29.4 (from normal)	+24.5 (from normal)
Sustained Handgrip [63]	Sustained handgrip for 3 minutes	MAP Range: 90-115	

Sustained Handgrip [64][65][66][68]	40% maximum contraction until fatigue	+45 to +50 (from normal)	+40 (from normal)
Valsalva Maneuver [67]	Breathe against closed mouth and nose for 15 seconds	-15 (from normal)	-15 (from normal)
Valsalva Maneuver [57]	Breathe against closed mouth and nose for 15 seconds	+6 (from the mean)	-4 (from the mean)

Table S3: Ranges in Systolic and Diastolic Blood Pressure from Baseline Across 10 Participants.

Subject	SBP range from normal (mmHg)	DBP range from normal (mmHg)
1	±23.0	±14.0
2	±41.6	±14.6
3	±35.2	±16.3
4	±36.8	±17.6
5	±30.2	±15.5
6	±30.5	±23.0
7	±30.4	±16.0
8	±66.1	±28.6
9	±43.2	±13.3
10	±44.1	±20.4
AVERAGE	±38.0	±19.0

Values in Supplementary Table 3 represent absolute deviations from baseline (Δ = (max–min)/2).

Table S4: Full Systolic and Diastolic Blood Pressure Ranges per Subject.

Subject	SBP range (mmHg)	DBP range (mmHg)
1	70.8 - 116.8	59.9 - 87.1
2	74.9 – 158.0	64.4 - 93.6
3	93.9 - 164.3	63.3 - 95.8
4	83.7 - 157.6	73.0 - 108.1
5	83.5 - 143.9	71.0 – 102.0
6	74.5 - 135.6	52.2 - 98.4
7	104.4 - 165.1	76.8 - 108.9
8	84.7 - 216.8	68.6 - 145.7
9	99.2 - 185.6	81.6 - 108.1
10	151.7 - 239.9	86.0 – 127.0

References

[26] Vysotskaya, N.; Will, C.; Servadei, L.; Maul, N.; Mandl, C.; Nau, M.; Harnisch, J.; Maier, A. Continuous non-invasive blood pressure measurement using 60 GHz-radar—A feasibility study. Sensors 2023, 23, 4111.

[29] Sel, K.; Osman, D.; Huerta, N.; Edgar, A.; Pettigrew, R.I.; Jafari, R. Continuous cuffless blood pressure monitoring with a wearable ring bioimpedance device. npj Digit. Med. 2023, 6, 59.

[48] Kireev, D.; Sel, K.; Ibrahim, B.; Kumar, N.; Akbari, A.; Jafari, R.; Akinwande, D. Continuous cuffless monitoring of arterial blood pressure via graphene bioimpedance tattoos. Nat. Nanotechnol. 2022, 17, 864–870.

[50] Wang, Y.-J.; Chen, C.-H.; Sue, C.-Y.; Lu, W.-H.; Chiou, Y.-H. Estimation of blood pressure in the radial artery using strain-based pulse wave and photoplethysmography sensors. Micromachines 2018, 9, 556.

[51] Li, S.; Wang, H.; Ma, W.; Qiu, L.; Xia, K.; Zhang, Y.; Lu, H.; Zhu, M.; Liang, X.; Wu, X.-E. Monitoring blood pressure and cardiac function without positioning via a deep learning–assisted strain sensor array. Sci. Adv. 2023, 9, eadh0615.

[57] Imholz, B.P.; Montfrans, G.A.V.; Settels, J.J.; Hoeven, G.M.V.D.; Karemaker, J.M.; Wieling, W. Continuous non-invasive blood pressure monitoring: Reliability of Finapres device during the Valsalva manoeuvre. Cardiovasc. Res. 1988, 22, 390–397.

[60] Yang, S. et al. Non-invasive cuff-less blood pressure estimation using a hybrid deep learning model. Opt. Quantum Electron. 2021.

[61] Kemmotsu, O. et al. Blood pressure measurement by arterial tonometry in controlled hypotension. Anesth. Analg. 1991.

[62] Hines, E.A.; Brown, G.E. The cold pressor test for measuring the reactivity of blood pressure. Am. Heart J. 1936.

[63] Hanson, P.; Nagle, F. Isometric exercise: cardiovascular responses in normal and cardiac populations. Cardiol. Clin. 1987.

[64] Petrofsky, J.S.; Lind, A.R. Aging, isometric strength and endurance, and cardiovascular responses to static effort. J. Appl. Physiol. 1975.

[65] Martin, C.E. et al. Autonomic mechanisms in hemodynamic responses to isometric exercise. J. Clin. Invest. 1974.

[66] Petrofsky, J.S. et al. Comparison of physiological responses of women and men to isometric exercise. J. Appl. Physiol. 1975.

[67] Parati, G. et al. Comparison of finger and intra-arterial blood pressure monitoring at rest and during laboratory testing. Hypertension 1989.

[68] Mukkamala, R. et al. Toward ubiquitous blood pressure monitoring via pulse transit time: theory and practice. IEEE Trans. Biomed. Eng. 2015.
